# Teaching Palliative Care Across Cultures: The Singapore Experience

**DOI:** 10.4103/0973-1075.76236

**Published:** 2011-01

**Authors:** Katrina Breaden

**Affiliations:** Flinders University, Adelaide, Australia

**Keywords:** Palliative care, Culture, Postgraduate education

## Abstract

Palliative care is a growing area of practice throughout the world and its promotion relies on adequately trained health care professionals. However, there are only a limited number of postgraduate academic courses or clinical training opportunities available, especially in resource challenged areas of the Asia Pacific region. This article outlines a creative endeavour between Flinders University, Adelaide Australia, the Singapore National Cancer Centre and the Asia Pacific Hospice and Palliative Care Network to provide an educational opportunity for students from the region. The strengths of the programme include its strong theoretical and evidenced-based framework, its multidisciplinary inclusiveness and its innovative and interactive teaching style. The main teaching challenge for the teaching team is to deliver culturally appropriate curricula to students from diverse cultural and linguistic backgrounds. This postgraduate programme is an important initiative for the region and for the development of future leaders and pioneers in the discipline.

## INTRODUCTION

Palliative care is a growing area of practice around the world and yet millions of people living with a life-limiting illness are denied access to it.

*While more than 100 million people annually would benefit from hospice and palliative care (including family and carers who need help and assistance in caring), less than 8% of those in need access it.*[1]

End of life care varies enormously from country to country, and even within countries. For example, while India is ranked 37^th^on the quality of death index (a measurement of end-of-life care in 40 countries), there are areas within this country such as Kerala where palliative care is well established.[2] The challenges of palliative care delivery in resource poor areas are several: there is a vast population that often has limited access to appropriate health care; there may be limited availability of opioids or government regulations that may control their use; and there may be cultural taboos or ethical imperatives against open and honest discussions around the dying phase. These challenges exist in many of the countries that make up the Asia Pacific region.

## THE COURSE

The World Health Organization states that palliative care “will enhance quality of life, and may also positively influence the course of illness.”[3] In order to achieve these outcomes, the education of future palliative care leaders within the region is vital. A program, recognized for its teaching excellence[4], has been developed by the Asia Pacific Palliative Care Collaborative.[5] Since 2006, the Graduate Certificate in Health (Palliative Care) has been offered in Singapore and attracts students from across the Asia Pacific region. The course consists of three main topic areas; palliative clinical management, communication at the end of life, and a four-week practicum placement to further develop the students’ level of palliative care knowledge and skills.

The Flinders University teaching team, in partnership with the Singapore National Cancer Centre and the Asia Pacific Hospice Network, delivers the course each year via a two-week intensive in May/June and again in October. The combination of these institutions and the local and international expertise of its members have ensured that the course continues to be highly regarded internationally.

The first student intake occurred in May 2006. Initially 16 students were chosen from 48 applications from nearly every country in South East Asia. The applicants’ backgrounds include nursing, social work, hospital administration, and medicine. Current and past students’ practice settings include pediatrics, oncology, general practice, palliative care, HIV medicine, intensive care, rehabilitation medicine, administration, acute and community care. The number of applications has risen each year and to date (2010), there have been a total of 91 students through the course. Students from Bangladesh, China, Hong Kong, India, Indonesia, Iran, Korea, Malaysia, Myanmar, Nepal, Philippines, Saudi Arabia, Singapore, Thailand, United Arab Emirates, and Vietnam have attended the course. Awareness of the course and international respect has grown and there continues to be a waiting list for subsequent intakes.

## TEACHING CHALLENGES AND OPPORTUNITIES

Teaching such a diverse cohort of students brings with it challenges and opportunities. The main challenge for the combined teaching team has been to deliver culturally appropriate and applicable curricula to students from diverse cultural and linguistic backgrounds. Many of the values and beliefs underpinning ethical principles such as truth-telling and patient/family autonomy differ between the regions; therefore, sensitive exploring of these areas is required to ensure that the information is relevant and applicable to each student’s practice and cultural context. The next challenge, mainly for the students, has been that the course is conducted in English and all students, whose undergraduate degrees were not taught in this language, must pass an International English Language test. This requirement means that many students wanting to enroll in the course are unable to do so, thereby limiting who has access to this form of education.

The differing levels of skills and practice knowledge of the students also poses a challenge for the teaching team; however, the use of small group activities and shared work tasks has enabled the creation of a level playing field. The students freely share their knowledge and skills with others, and, in the process, they learn from each other and not just from faculty.

The delivery of the course in Singapore has also created several opportunities. Interacting with clinicians from differing practice contexts and countries has assisted in the process of internationalizing the curricula for the benefit of all our students-nationally and internationally. An additional opportunity this course has provided is in the area of fostering and inspiring students to become lifelong learners. The development of lifelong learning strategies is desirable in all students, irrespective of their practice contexts. There are five main approaches to teaching that have fostered these qualities: (1) the design and delivery of teaching materials that show respect for an understanding of vast cultural differences, (2) the use of small group activities to explore content areas, (3) required prereading and assignment preparation prior to attendance at the intensives, (4) frequent support from the teaching team via email and Skype, and (5) support and contact with clinicians and academics from within the region.

There has also been the opportunity to work with different teaching and learning styles. These differences, present in both students and faculty, are discussed openly and used in class to advance learning. By virtue of the critical aspects of the program, students are encouraged to question teachers and challenge their own long held professional beliefs that may rest on very little evidence. A variety of approaches to teaching are used to advance such critical and lateral thinking. For example, the teaching team encourages students to create methods and models of palliative clinical practice that are culturally and regionally appropriate for their local community and to apply to them a critical framework of analysis.

An additional teaching opportunity has been in the area of critical thinking development. The palliative care programs at Flinders University have a strong tradition of critical thinking, and, with guidance from teachers, students are academically rewarded for questioning and finding answers for themselves. The intercultural teaching and learning resulting from the teaching opportunity has presented a rich medium for growth of both students and teachers. These approaches to teaching differ from other approaches in palliative care offered internationally in that they: (1) are multidisciplinary, (2) use an interactive and open teaching style, and (3) offer clinical placements with joint faculty supervision. This unique combination of teaching strategies has enabled the students to become more effective teachers themselves, vital for the continued development of the discipline.

In order to influence, motivate, and inspire palliative practitioners, the teaching team has developed innovative approaches to teaching and learning. What is particularly innovative about the Singapore program is the opportunity to collaboratively teach with local experts, the use of a team teaching approach and the development and application of a critical framework from which to question clinical decision making in local contexts.

## INSPIRING LEARNING

The students’ participation in the Graduate Certificate has inspired them in ways that were perhaps unforeseen at the beginning of the project. For example, one student was appointed as medical director after completing the course and went on to revise the entire inpatient hospice system of her hometown in India. Another student became involved in planning and liaison with government agencies for the delivery of pediatric palliative care services in Jakarta. A new home care program was established in Manila, and innovative palliative education programs were set up in Rajasthan and Thailand. The graduates have found the confidence and the courage to become pioneers and leaders in their respective fields. A student from Sichuan China highlights, in the following extract, her enthusiasm for ongoing learning. She learnt how to learn, a necessary foundation for continued palliative practice.

I’ve [sic] have practiced palliative medicine since my graduation in 2005. However, I have not had the opportunity to receive formal professional training in palliative care in China because it is quite a new discipline. The trip to Singapore was a wonderful opportunity to learn more about palliative care. I was so honored to attend lectures given by the teachers from Flinders University. Most important, I was able to learn how to learn. There is an old Chinese saying that, “teaching people to fish is better than giving people fish”. My most respected teachers taught me a very good lesson on how to “fish”[6]

The above quote is not the only support and praise the teaching team has received; many others have also commented on the value it has added to their working lives. A student from the Philippines was inspired to add new clinical methods to his treatment toolbox:

I could say that the course gave my practice a “facelift.” I am now more confident because I gained plenty of new knowledge from the course. It inspired me to apply methods that were alien to our setting such as subcutaneous administration of medicines including infusions for hydration. To borrow from one of my teacher’s sayings, I am now armed with a lot more weapons, i.e., choices in dealing with palliative care patients (including psychosocial skills).

A Singaporean doctor was motivated to become a leader in his community:

The course has helped me to consolidate my calling as a palliative care physician and I am now the Head of a new hospice in Singapore-thus I am closer to my dream of gathering resources from the health care sector, the charity sector and the Church, and channeling them into the ministry for the terminally ill.

And finally, as a result of the course, one doctor has changed his career direction:

“*I decided to shift from Lecturer in Surgery, to become a full-time palliative care doctor.”[7]*

## CONCLUSION

The teaching of this program is an important initiative within the region, and both the teaching team and the students learn from each other. To see the extraordinary services being provided to people living with a life-limiting illness in resource-challenged areas has been extremely insightful. The palliative practitioners devote substantial amounts of personal time and absence from their paid employment to pursue this educational opportunity. Their dedication and commitment to the discipline has been humbling to witness. The team’s innovative approaches to teaching and learning continue to influence, motivate, and inspire a whole cohort of future-directed palliative practitioners. Their enthusiasm for ongoing learning is indeed inspiring, and it is hoped that the course will sustain the development and growth of palliative care within the Asia Pacific regions for years to come.
